# The spreading of parasites by human migratory activities

**DOI:** 10.1080/21505594.2020.1809963

**Published:** 2020-08-30

**Authors:** Dietmar Steverding

**Affiliations:** Bob Champion Research and Education Building, Norwich Medical School, University of East Anglia, Norwich, UK

**Keywords:** Protozoan parasites, helminths, arthropods, spread, range expansion, migration, trade

## Abstract

The global spread of parasites is unquestionably linked with human activities. Migration in all its different forms played a major role in the introduction of parasites into new areas. In ancient times, mass migrations were the main causes for the spread of parasites while in the recent past and present, emigration, immigration, displacement, external and internal migration, and labor migration were the reasons for the dispersal of parasites. With the advent of seagoing ships, long-distance trading became another important mode of spreading parasites. This review summarizes the spread of parasites using notable examples. In addition, the different hypotheses explaining the arrival of *Plasmodium vivax* and soil-transmitted helminths in pre-Columbian America are also discussed.

## Introduction

Ever since *Homo sapiens* emerged in eastern Africa some 200,000 to 100,000 y ago [1], humans peopled the world until 15,000 y ago they inhabited all continents apart from Antarctica. On their journey, humans brought some of their parasites with them (heirloom parasites) while others were acquired from animals with which they had come in contact during migration (souvenir parasites). Because of the different climate conditions in the different regions of the world, humans would have carried along only a few parasite species. This would include only permanent parasites or those with free-living stages, which can become infectious within a short period of time even in cold temperature, or those with intermediate hosts or vectors, which are locally available and in which infectious live-cycle stages can develop under the regional climate conditions. Parasites that have been most likely spread worldwide during prehistoric human migration are the head lice *Pediculus humanus*, the pinworm *Enterobius vermicularis*, the whipworm *Trichuris trichiura*, the roundworm *Ascaris lumbricoides*, the threadworm *Strongyloides stercoralis*, the hookworms *Ancylostoma duodenale* and *Necator americanus*, and the tapeworms *Taenia saginata* and *Diphyllobothrium latum*. Evidence for this is the demonstration of these parasite species in archeological specimens (e.g. coprolites and mummies) of prehistoric people in North and South America [2–6]. Likewise, there are indications that *P. humanus* (clade A), *E. vermicularis*, and *A. lumbricoides* were present in Australasia before the first Europeans arrived [7,8].

By 8,000 BCE, humans started to switch from a nomadic, hunter-gatherer lifestyle to a settled, agricultural way of life. This transition is characterized by the domestication of livestock, which brought humans in close contact with parasites harbored by their farmed animals. With the construction of seagoing ships by 4,000 BCE, humans became more mobile and established long-distance trading routes. By 3000 BCE, climate change, poor harvests, and population pressure were the reasons for large mass migrations. All these events led to the spread of parasites, which, until then, had only a regional distribution. Other human activities like conquest, war, displacement, relocation, slavery, emigration, immigration, and travel contributed also to the dispersal of parasites. This second period of parasite spreading did not only happen in the past but also continued in modern times. This review provides examples of the introduction of parasite species into other parts of the world as a result of human migratory activities.

## Endoparasites

### Malaria parasites

The origin of *Plasmodium falciparum* (Table 1), the causative agent of severe human malaria, in the Americas is controversial. Some studies suggest that the parasite was recently introduced into the New World during slave trade, Spanish conquest, and European immigration [9]. Other evidence suggests that *P. falciparum* was already present in South America in pre-Columbian times [9]. New evidence indicates that *P. falciparum* evolved following a zoonotic transfer of a parasite from gorillas about 50,000 y ago and emerged from a bottleneck of a single parasite around 4,000–6,000 y ago [10,11]. Based on these new findings it seems unlikely that *P. falciparum* was part of the original parasite fauna of the Americas. Thus, the only remaining possibility for the presence of *P. falciparum* was the introduction of the parasite into the New World in post-Columbian times. This scenario is supported by genetic studies analyzing microsatellite and SNP polymorphisms and mitochondrial DNA diversity in *P. falciparum* populations indicating that enslaved Africans were likely the main carriers of this malaria species into America [12,13].

**Table 1.** Information about protozoan parasites spread by human migratory activity.

Name	Life cycle type	Vector	Origin	Current distribution
**Plasmodiidae**				
*Plasmodium falciparum*	indirect	*Anopheles* sp.	Western Africa	worldwide but mainly tropical and subtropical
*Plasmodium vivax*	indirect	*Anopheles* sp.	Central Africa	worldwide between latitudes 16° N and 20° S
**Trypanosomatidae**				
*Leishmania infantum*	indirect	*Lutzomyia* sp. *Phlebotomus* sp.	East Africa	Mediterranean region, Middle East, East Africa, China, Latin America
*Trypanosoma evansi*	indirect	*Tabanus* sp. *Stomoxys* sp.	Western Africa	North Africa, Near and Middle East, Central and South Asia, Latin America
*Trypanosoma equiperdum*	direct	-	Eastern Africa	Mediterranean region, North and South Africa, Asia, Latin America
*Trypanosoma vivax*	indirect	*Glossina* sp. *Tabanus* sp.	sub-Saharan Africa	tropical Africa, South America, Caribbean
*Trypanosoma brucei*	indirect	*Glossina* sp.	sub-Saharan Africa	sub-Saharan Africa
*Trypanosoma cruzi*	indirect	*Triatoma* sp. *Rhodnius* sp. *Panstrongylus* sp.	South America	South and Central America

**Figure 1.**
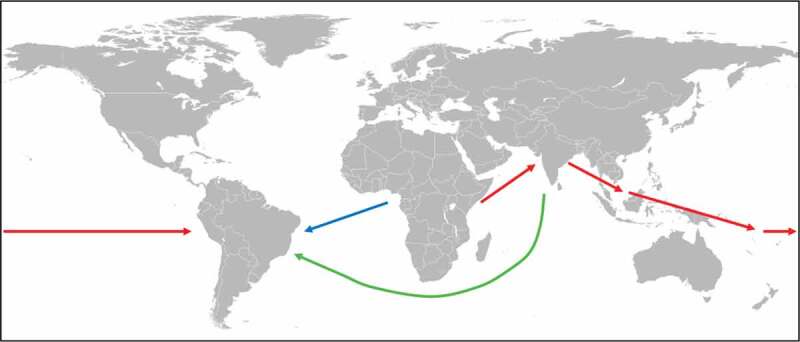
Spreading of *P. vivax.*

As for *P. falciparum*, the arrival of the less deadly human malaria species *P. vivax* (Table 1) in the New World is also controversial. Previous studies suggest that *P. vivax* emerged in Southeast Asia after crossing the species barrier from a macaque to human [14]. However, new research points to an origin of *P. vivax* in Africa from a strain that was able to infect both humans and apes [15–17]. The parasite was largely eliminated in Africa by the spread of the Duffy-negative mutation while a single lineage spread through Asia and Europe representing the current human-infecting *P. vivax* species [15,17]. Recent genetic studies investigating the mitochondrial DNA diversity in *P. vivax* populations found significant genetic contribution from African and South Asian lineages with some additional genetic input from Melanesian lineages to the *P. vivax* strains of the Americas [13]. This finding suggests that the extant African and South Asian *P. vivax* populations represent the major contributors to the New World lineages of *P. vivax* and were introduced in post-Columbian times most likely by slaves from Africa and migrants from Asia (Figure 1). In addition, Australasian people may have brought *P. vivax* from the Western Pacific to the Americas in pre-Columbian times (Figure 1). It can be assumed that a malaria parasite causing recurrent infections might survive long-range ocean crossings [18]. The new findings also indicate that the founding population entering the American continent via the Behring land bridge did not bring *P. vivax* to the New World, and that this parasite was first introduced later by Melanesian seafarers, but some time before the Europeans arrived. This suggestion is corroborated by the detection of *P. vivax* antigens using chromogenic immunohistochemistry in 3,000 to 600 y old South American mummies [19].

New research indicates that *P. vivax* originated in Africa and was spread to Asia and Australasia, from where the parasite was spread to South America in pre-Columbian times (red arrows) [13]. In post-Columbian times, *P. vivax* was introduced into Latin America from West Africa by slaves (blue arrow) and from Asia by migrants (green arrow) [13].

The spread of malaria due to military conflicts has been a constant theme throughout the entire human history until modern times. A more recent example for this is the introduction of *P. vivax* malaria in Berlin at the end of the Second World War. In the summer of 1946, *P. vivax* was spread among the inmates of a prisoner-of-war camp near Lake Tegel by German soldiers who were also interned in the detention facility upon their return from Southeast Europe and Africa. Subsequently, the parasite was quickly spread to local residents with more than 500 cases recorded [20,21].

Malaria is also the most frequent imported parasitic infection in non-endemic countries. Over the last 50 y, imported malaria has continuously increased due to growing international travel and migration. For example, between 1972 and 2000, the number of imported malaria cases rose from 1,500 to 15,500, most of which were reported by Western European countries with France, Germany, and the United Kingdom counting for more than 70% of all cases [22]. However, since 2004 the number of malaria cases imported to the United Kingdom has remained unchanged at approximately 1,700 cases per year [23]. Most of the imported malaria cases were caused by *P. falciparum* acquired during travel to West Africa [24]. However, over time the epidemiological characteristics of imported malaria cases have changed. Whereas previously malaria was mainly imported to non-endemic countries by returning travelers, the last 10 y have seen a continuous increase of imported malaria by immigrants and settled immigrants who traveled to visit relatives and friends still living in their country of origin after their return [22].

### Trypanosomatids

With the exception of *Leishmania chagasi*, all other *Leishmania* species already inhabited the different continents long before modern humans had evolved [25]. *Leishmania chagasi* is the causative agent of visceral leishmaniasis in South America and its origin has been widely debated [26,27]. Meanwhile, it seems clear that *L. chagasi* is identical with *L. infantum* (Table 1) and was introduced into the New World in post-Columbian times. Evidence for this comes from Bayesian phylogenetic analysis showing that *L. chagasi* clusters with the Portuguese *L. infantum* clade [28]. A bottleneck signature indicates that the parasite was introduced in South America about 500 y ago, probably by European settlers, and their dogs [28].

*Trypanosoma evansi, T. equiperdum*, and *T. vivax* (Table 1) are examples of animal pathogenic parasites that were spread by humans throughout the world. All three trypanosome species belong to the Salivarian group which evolved about 35 million years ago in Africa [29]. *Trypanosoma evansi* is the etiological agent of Surra in livestock animals, particularly in camelids and equids. The parasite is mechanically transmitted by biting flies such as tabanids and stomoxes. It was suggested that *T. evansi* was introduced beyond Africa by the ancient Egyptians during military campaigns in the Middle East [30]. From there, the parasite spread further eastwards and by the eighth century BCE had reached India [30]. The parasite was introduced into South America first in Colombia in the sixteenth century by Spanish conquistadors, and later in Brazil in the nineteenth century [31]. In Latin America, *T. evansi* is also transmitted via the common vampire bat *Desmodus rotundus*, which not only acts as biological vector but also as a host and reservoir for the parasite [32]. *Trypanosoma equiperdum* is the pathogen causing dourine in horses, and unlike other trypanosomes, is not transmitted by an insect vector, but directly from one animal to another during sexual contact. Because of the mode of transmission, *T. equiperdum* was easily spread worldwide through horse trade [31]. After the Second World War, *T. equiperdum* was eradicated from North America and Western Europe by systematic screening and control [31,33]. *Trypanosoma vivax* is one of the three African trypanosomes responsible for nagana disease in cattle. In Africa, *T. vivax* is mainly cyclically transmitted by tsetse flies, but also mechanically by biting flies. According to Curasson [34], *T. vivax* was introduced into Latin America with a shipment of zebu cattle from Senegal to French Guyana, Guadeloupe, and Martinique in 1830. However, the parasite may have been introduced already in the eighteenth century, as cattle were shipped directly from Africa or indirectly via Caribbean islands as early as 1733 [35]. As there are no tsetse flies in the New World, *T. vivax* is transmitted mechanically by tabanids and became permanently established all over South America.

It can be reasonably assumed that *T. brucei* (Table 1), the causative agent of African sleeping sickness, must also have crossed the Atlantic with the slave trade. This can be inferred from accounts of ship doctors and medical officers employed by slave-trade companies [29]. However, *T. brucei* could never become established in South America because tsetse flies, the required vector for transmission of the protozoan parasite, are absent from the New World.

*Trypanosoma cruzi* (Table 1), the pathogen responsible for Chagas disease, is an example of a parasite that is spread by migrants from Latin American endemic countries to other parts of the world. Recent evidence suggests that *T. cruzi* evolved from bat trypanosomes about 7–10 million years ago in South America [36]. The parasite is transmitted via the feces of triatomine bugs (kissing bugs or conenose bugs) and by other modes including blood transfusion, organ transplantation, breastfeeding, congenital transmission, and ingestion of contaminated food and drinks. In recent times, Chagas disease has become increasingly a global health problem as the estimated number of people infected with *T. cruzi* outside Latin America (mainly North American and European countries) is more than 400,000 [37,38]. Although any spread of *T. cruzi* in non-endemic countries is unlikely as most triatomine species are restricted to tropical areas in Latin America, enzootic infection of wild animals and autochthonous infections of humans have been reported from some southern states of the USA [39,40]. In addition, with the tropicopolitan distribution of *Triatoma rubrofasciata* [41], a kissing bug species that transmits *T. cruzi* in the Americas, the scene is set for potential transmission of Chagas disease outside the New World, if South Americans would immigrate to tropical Asian and African countries [42]. Regarding the global spread of *T. rubrofasciata*, see section on Ectoparasites below.

### Flatworms

**Figure 2.**
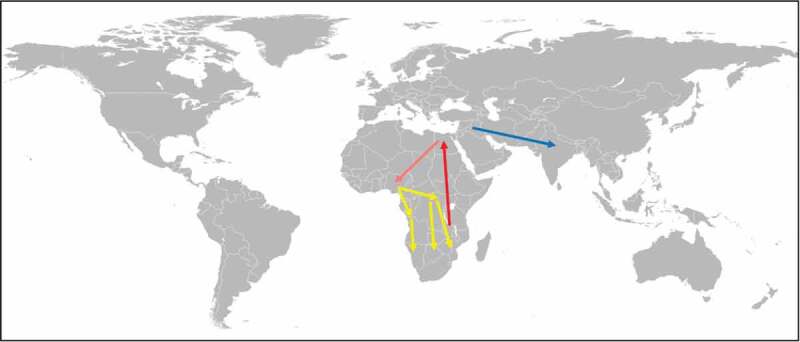
Spreading of schistosomes (*S. mansoni* and *S. haematobium*) and *A. duodenale* by mass migration.

Schistosomes are blood-dwelling flukes that, based on molecular data, have evolved in Asia and have dispersed into Africa by migration of their mammalian definitive and snail intermediate hosts [43]. Initially, schistosomes were probably parasites of animals and there is evidence that human schistosomiasis evolved as a zoonosis in the region of the African Great Lakes [44]. From there, it seems that the parasites spread to Egypt by the import of monkeys and slaves [44]. Whether the further spread of schistosomes (*S. mansoni* and *S. haematobium* (Table 2)) from Egypt to West Africa and subsequently to Central and South Africa was the results of the Yoruba and Bantu migration, respectively, is uncertain (Figure 2) [45]. However, it can be taken as certain that *S. mansoni*, the etiological agent of intestinal schistosomiasis in humans, was introduced in post-Columbian times into Latin America presumably by enslaved Africans. This suggestion is supported by phylogeographic analyses of mitochondrial DNA indicating that the genetic diversity of New World *S. mansoni* strains comprises only seven closely related haplotypes with West African affinities [46,47]. More recently, schistosomes have been introduced into non-endemic regions by immigrants and migrant workers. An interesting example in this context is the founding of the state of Israel in 1948. Within a few years 500,000 immigrants from schistosomiasis endemic regions from Near Eastern countries had entered Israel, of which 6–8% were infected with schistosomes [48]. Many immigrants settled along the river Yarkon near Tel Aviv where in 1951 nineteen schoolchildren contracted *S. mansoni* while swimming in the river [48]. In 1955, about 100 schoolchildren contracted *S. haematobium* after bathing in a water-storage reservoir in the Beit-She’an valley [48]. Further transmission of *S. mansoni* was stopped as its snail vector *Biomphalaria alexandria* was successfully eradicated. Also, transmission of *S. haematobium* has not been seen since, although its snail vector *Bulinus truncatus* is still widespread in Israel. An example of the introduction of a parasite by migrant workers is the spread of *S. mansoni* at the Wonji Sugar Estate in the upper Awash valley in Ethiopia [49]. Although from the beginning every effort was made to ensure that none of the migrant workers to be employed was infected with schistosomes, in 1964, 10 y after the sugar estate had been established, first cases of *S. mansoni* infection among the laborers were recorded. Previously, both *S. mansoni* and the host snail were unknown in the area. The parasite was introduced by migrant workers from the north central highlands of Ethiopia where *S. mansoni* was endemic. By 1980, the prevalence of intestinal schistosomiasis had risen to 20% in the area [50] and in 1988 the prevalence of *S. mansoni* infections in children in one of the labor camps at the Wonji estate reached 82% [51]. The main reason for the spread of *S. mansoni* in the region was the poor maintenance of sewage and hygiene facilities at the Wonji estate labor camps with latrine pipes leaking directly into canals, so that the general public living outside the sugar plantation was also affected. Another interesting case is the outbreak of urogenital schistosomiasis on the island of Corsica in the summer of 2013 when more than 120 local people and tourists contracted the parasitic disease [52]. All affected persons had never been to a schistosomiasis-endemic area and had been swimming in the Cavu River which harbored many *B. truncatus* snails. Molecular characterization of eggs or hatched miracidia recovered from 12 patients showed infection with *S. haematobium, S. haematobium*/*S. bovis* hybrids and *S. bovis*. Sequence data analysis indicated that the parasites must have been introduced by individuals who contracted the schistosomes in Senegal. This case shows how easily and rapidly schistosomes can be introduced and spread into new areas provided vector host snails are present.

**Table 2.** Information about flatworms spread by human migratory activity.

Name	Life cycle type	Intermediate host(s)	Origin	Current distribution
**Schistosomatidae**				
*Schistosoma mansoni*	indirect	*Biomphalaria* sp.	East Africa	Africa, Middle East, parts of South America and the Caribbean
*Schistosoma haematobium*	indirect	*Bulinus* sp. *Physopsis* sp.	East Africa	Africa, Middle East
**Fasciolidae**				
*Fasciola hepatica*	indirect	*Lymnea* sp.	Eurasia	worldwide
*Fascioloides magna*	indirect	*Fossaria* sp. *Stagnicola* sp. *Galba truncatula* *Radix perega*	North America	North America, Europe
**Opisthorchiidae**				
*Opisthorchis viverrini*	indirect	1^st^: *Bithynia* sp. 2^nd^: Cyprinidae	Southeast Asia	Thailand, Cambodia, Laos
**Taenidae**				
*Taenia solium*/cysticercosis^a^	indirect	pigs	sub-Saharan Africa	worldwide

^a^In cysticercosis, the transmission of *T. solium* is direct from human to human via ingestion of eggs released by humans infected with the tapeworm, and thus humans are final and intermediate host at the same time.

African schistosomes have evolved in the region of the African Great Lakes, from where they have been spread along the Nile (red arrow) [44]. It is likely that the schistosomes have been dispersed to West Africa in the course of the Yoruba mass migration (orange arrow) and further to Central and South Africa in the course of the Bantu mass migration (yellow arrows) [45]. Originated in the Mediterranean region, *A. duodenale* has been introduced into North India in the course of the Aryan mass migration (blue arrow) [45].

Liver flukes (fasciolids) are parasites of herbivores, but they can also cause disease in humans. Molecular phylogenetic analysis suggests that fasciolids originated in African proboscideans, and later radiated in Eurasian herbivores [53]. *Fasciola hepatica* (Table 2), the common liver fluke, is likely of Eurasian origin from where the parasite was spread with infected livestock to other continents and where it adapted to other lymnaeid intermediate host snail species [53,54]. This is evident from molecular clock estimations based on ITS sequences indicating that *F. hepatica* was recently introduced from Europe into South America by the import of livestock at the time of the Spanish colonization [54]. An interesting case of reverse introduction from the New World into the Old World is the large American Liver Fluke, *Fascioloides magna* (Table 2). Probably proboscideans brought a fasciolid with them to the Nearctic in prehistoric times, where the parasite evolved into *F. magna* after adapting to cervids following the extinction of proboscideans in North America [53]. *Fascioloides magna* was at least twice introduced into Europe with imported game animals [55]. The parasite was introduced with wapiti (*Cervus canadensis*) brought from original habitats in North America to Italy in the nineteenth century and to Bohemia at the beginning of the twentieth century. Meanwhile, *F. magna* has become established in Slovakia, Austria, Germany, Hungary, Croatia, and Serbia, spreading along the Danube River [55].

An example for the dispersal of a parasite by internal migration is the Southeast Asian liver fluke *Opisthorchis viverrini* (Table 2) in Thailand. Whereas in the 1950s the prevalence of *O. viverrini* in the Northeast region was extremely high (locally up to 80–100%), only sporadic cases of opisthorchiasis were registered in the Central region from which it was unclear whether these few cases were due to autochthonous infections [45,56]. Between 1955 and 1980, hundreds of thousands of people, many of them infected with *O. viverrini*, migrated from the Northeast region to the Central region to find a better livelihood [45]. The consequence was that within these 25 y the prevalence of *O. viverrini* in the Central region increased on average to over 14% [45]. Meanwhile, it is recognized that *O. viverrini* has become endemic in the Central region as with the presence of the first and second intermediate hosts (freshwater snails of the genus *Bithynia* and freshwater fish of the family Cyprinidae) the conditions to complete the life cycle of the parasite were met [45]. Although the nationwide prevalence of opisthorchiasis has decreased since the 1980s due to the implementation of helminthiasis control programs, the problem of migrants moving from the still highly endemic Northeast region to other neighboring areas still continues leading to the establishment or reestablishment of local transmission of *O. viverrini* [57].

A curious case of parasite spreading is the introduction of the pork tapeworm *Taenia solium* (Table 2) into Western New Guinea [58]. Between 1973 and 1976, an unprecedented increase in hospital admission due to high-degree burns among the native Ekari people living in the central highlands of the province Papua was reported [59]. The burns resulted from epileptic seizures while the tribal people were sleeping causing them to fall into fire places. Soon it was established that the epileptic seizures were due to neurocysticercosis caused by the larval stage of *T. solium*. As New Guinea was free of *T. solium* until then, the question was, how the parasite was brought to the island? After the Dutch left Western New Guinea in 1969, the United Nations allowed the inhabitants to join Indonesia. However, as the people were undecided, the Indonesian government dispatched soldiers from Bali, where taeniasis was quite common at that time, to create a *fait accompli*. Since pigs play an important role in the ritual life of the Etari, the Indonesian government tried to appease them with a present of pigs which, unfortunately, were infected with *T. solium* cysticerci [58]. Cysticercosis is still endemic in Central Papua and has meanwhile spread to other regions of the province [58,60].

### Nematodes

**Figure 3.**
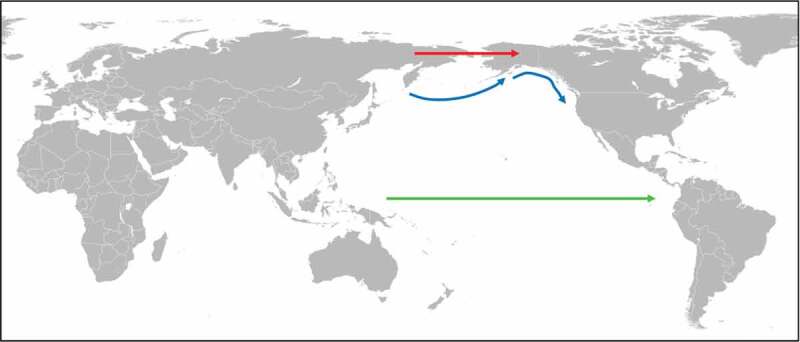
Introduction of soil-transmitted helminths into the Americas.

Although paleoparasitological evidence dating back to 9,000 y ago show that the soil-transmitted helminths *A. duodenale, N. americanus, T. trichiura, A. lumbricoides*, and *Strongyloides stercoralis* (Table 3) were present in ancient inhabitants of North and South America, these parasites could not have been introduced into the New World with the first humans crossing the Bering Land Bridge [3,5,6,19]. This is because transmission of these helminths depends on the maturation of eggs or larvae released into the environment. However, in order to reach infectivity, eggs and larvae need at least moderate temperature and high soil moisture, conditions that could not have been met in Beringia. In addition, soil-transmitted helminths have never been reported in arctic and subarctic indigenous people living traditional lifestyles [5]. In contrast, the pinworm *E. vermicularis* does occur in native populations of the arctic as its eggs are not dependent on climate conditions. *Enterobius vermicularis* eggs are already infective as soon as they are released and are transmitted via the fecal-oral route usually within family groups [5]. In the case of *S. stercoralis*, it might be possible that this helminth was brought to the New World by migration through Beringia as autoinfections can maintain the parasite in an individual for a lifetime. However, transmission to a new host would still require that the parasite completes its lifecycle in the environment, which would not be possible under the arctic climate conditions. Thus, how did soil-transmitted helminths enter the New World? A possible explanation would be alternative migration routes to the New World in prehistoric times. One such alternative route could have been a boat-supported coastal migration pathway which could have provided environmental conditions suitable for eggs and larvae of soil-transmitted helminths to mature [61]. This possibility is supported by recent findings indicating that Alaska’s coast was clear of ice from about 17,000 y ago [62], which would have made it feasible for early humans to move south along the shoreline spreading parasites into the New World. Another alternative route could have been a trans-oceanic crossing by people from the West Pacific to the Americas [5]. As such trans-pacific migration would have happened relatively quickly, intestinal parasites could have easily survived the crossing. Support for this hypothesis comes from a recent genome-wide analysis revealing that at least three South American indigenous peoples descended from a native American founding group that shares more ancestry with indigenous people from Australia, New Guinea, and the Andaman Islands than with Eurasians or other Native Americans [63]. Thus, it is plausible that soil-transmitted helminths have been spread to the New World by a different route during a second migratory wave after the Clovis migration across Beringia (Figure 3).

**Table 3.** Information about nematodes spread by human migratory activity.

Name	Life cycle type	Intermediate host(s)/vector	Origin	Current distribution
**Ancylostomatidae**				
*Ancylostoma duodenale*	direct	-	North African Mediterranean region	worldwide but predominately in the Middle East, North Africa and southern Europe
*Necator americanus*	direct	-	presumably in Africa	worldwide but predominately in the Americas and Australia
**Trichuridae**				
*Trichuris trichiura*	direct	-	presumably in Africa	worldwide but more frequent in tropical areas
**Ascariidae**				
*Ascaris lumbricoides*	direct	-	presumably in Africa	worldwide but predominately in tropical and subtropical areas
**Strongyloidae**				
*Strongyloides stercoralis*	direct	-	presumably in Africa	worldwide in tropical and subtropical areas
**Onchoceridae**				
*Onchocerca volvulus*	indirect	*Simulium* sp.	Africa	Africa, Middle East, Latin America
**Filaridae**				
*Wuchereria bancrofti*	indirect	*Aedes* sp. *Culex* sp.	Malay Archipelago	worldwide in tropical areas
*Mansonella perstans*	indirect	*Culicoides* sp.	most likely in Africa	West and Central Africa, South America
*Loa lao*	indirect	*Chrysops* sp.	most likely in Africa	West and Central Africa
**Dracunculoidea**				
*Dracunculus medinensis*	indirect	*Cyclops* sp.	presumably in Africa	sub-Saharan Africa, Ethiopia, Middle East, India
**Metastrongylidae**				
*Angiostrongylus cantonensis*	indirect	snails, slugs, crabs, shrimps	southern Asia	Southeast Asia, Pacific Basin, Africa, Caribbean

It is unlikely that soil-transmitted helminths (*A. duodenale, N. americanus, T. trichiura, A. lumbricoides*, and *S. stercoralis*) have been introduced into the New World by humans migrating through Beringia (red arrow) because the harsh climate conditions of this route would have been detrimental for the development of their free-living life cycle stages [5]. Instead, soil-transmitted helminths could have been introduced into the New World via costal migration (blue arrows) and/or trans-pacific migration (green arrow) in pre-historic times [5,61–63].

*Ancylostoma duodenale* is also an example of a parasite that had been spread by mass migration. The hookworm is thought to have originated in the Mediterranean region and was probably brought to northern India by the Aryans in the twelfth century BCE. The Aryans were a Central Asian pastoral tribe who from the sixteenth century BCE onwards were migrating to northern India. The possibility that the Aryans have introduced *A. duodenale* into northern India is supported by observation made in the 1920s (Figure 2). According to Darling [64], the incidence of *A. duodenale* infection among the people living in the north of the subcontinent was up to 80% while among the native Tamils living in the south, where the other hookworm species *N. americanus* was dominant, it was only about 2%.

The intestinal bloodsucking hookworm *A. duodenale* is probably the only parasite that became an industrial occupational hazard. The discovery of *A. duodenale* as a workplace risk began with the construction of the Saint Gotthard Tunnel between Switzerland and Italy in 1871. By 1879/80, large number of workers became so anemic that they were unable to continue to work [65]. At first, it was thought that the workers were suffering from “miner’s disease,” a condition known for decades affecting mineworkers in different types of pits all over Europe. However, by 1881 it was clear that miner’s disease was actually caused by *A. duodenale* [66]. Subsequently, the hookworm was reported in miners worldwide with infection rates close to 100% in some places [65,67]. In contrast, surface mine employees were usually not affected by the infection indicating that the hookworm problem was intrinsically linked with the underground workings [67,68]. This poses the question of how it was possible that a tropical/sub-tropical parasite could manifest itself so dramatically in mines throughout the world? The answer to this lies in the working conditions of miners at that time. First, the poor sanitation conditions (lack of proper latrines and sewage disposal) meant that miners relieved themselves almost anywhere in the mines. Second, the warm and damp conditions of mines were favorable conditions for the development of hookworm larvae. The hot environment of deep mines is due to the geothermal gradient, i.e., Earth’s temperature increases with depth (about 2–3°C per 100 m). But also in deep buried long tunnels like the Saint Gotthard Tunnel (15 km long with a maximum depth of 1700 m), it is likely to encounter temperatures more than 35°C. The elevated temperature in turn leads to the evaporation of more groundwater and mine water causing an increase in humidity. In addition, the practice of sprinkling to prevent coal dust explosion resulted in an increase in hookworm prevalence in many coal mines. In practice, this meant that mud was building up everywhere that was carried about throughout mines including the rungs of ladders. Thus, if the mud was contaminated with hookworm larvae, it was almost inevitable for miners not to get infected. Important for the spreading of *A. duodenale* throughout mines all over the world was the high mobility of mineworkers. For example, in 1913, the German Ruhr region recorded that within the previous 12 months 69% of coal miners left their pits while 78% were newly hired [69]. In contrast, in parts of England miners were constantly recruited from various regions of the world where hookworm infections were endemic [68]. However, it seems also very likely that ancylostomiasis had been within the mining community for centuries, from ancient Egyptian slave pits to medieval European iron mines, and early modern mining operations, constantly passing hookworm infections from one generation of mineworkers to the next [67]. In the end, *A. duodenale* was successfully eliminated from mines worldwide by applying hygienic, preventative, and therapeutic measures developed and recommended as a consequence of the hookworm epidemic at the Saint Gotthard Tunnel [65].

The filarial worms *Onchocerca volvulus, Wuchereria bancrofti, Mansonella perstans,* and *Loa loa* (Table 3) are further examples of parasites that were brought to the New World by infected slaves in post-Columbian times. In the case of *O. volvulus*, the parasite causing river blindness in humans, analysis of a tandemly repeated DNA sequence family revealed that the American strains are indistinguishable from the African savannah strains indicating that the parasite was recently introduced into Latin America [70]. Although *Simulium damnosum*, the main vector of *O. volvulus* in West Africa, is not found in the New World, other suitable *Simulium* species were present helping to spread the parasite throughout Latin America. However, as the different *Simulium* species do not cover wide geographical areas, *O volvulus* occurs only in relative small and isolated foci in the New World [71]. *Wuchereria bancrofti*, the major cause for lymphatic filariasis, is an example of a parasite that was spread throughout tropical and subtropical regions of the world by human migration. Probably originated in the Malay Archipelago, *W. bancrofti* was first dispersed throughout Southeast and East Asia about 50,000 y ago. Austronesians most likely introduced *W. bancrofti* into Madagascar at about 1,500 to 1,800 y ago from where later migrations spread the parasites to continental Africa. Finally, *W. bancrofti* was spread from West Africa to the New World during transatlantic slave trade in post-Columbian times. Subsequently, the parasite was further distributed in Latin America by the migration of people within the colonies. For example, *W. bancrofti* was introduced into Costa Rica in 1871 by infected Jamaicans who came as labourers to help building the railways between Puerto Limón and San José [72]. However, wuchereriasis did not expand to rural areas, and ever since its introduction remained a problem in Costa Rica of urban areas of Puerto Limón [72]. The geographic origin and the timing of the global wide dispersal of *W. bancrofti* was recently corroborated by whole genome amplification analyses [73]. In contrast to other filarial worms, *M. perstans* causes only minimal pathology with few disease symptoms. Recent phylogenetic analysis of ribosomal and mitochondrial DNA sequences revealed a close relationship between *M. perstans* strains from South America and Africa suggesting that this filarial worm was also introduced into the New World in post-Columbian times as a consequence of slave trade [74]. *Loa lao*, the African eyeworm, has repeatedly been introduced into Latin America in the recent past but could never have established itself in the New World [75]. The introduction of *L. loa* into the New World is evident from its first description by the French Surgeon Mongin who saw the parasite in the eye of a slave from the Caribbean in 1770 and from the observation by the French ship surgeon Francois Guyot who noticed recurrent ophthalmia in slaves on their way from Africa to America and successfully removed the worm from one victim in 1778 [76].

*Wuchereria bancrofti* also provides an example of how the expansion of agriculturally cultivated land can increase the prevalence of a parasitosis. Between 1903 and 1937, the Davao region on the island of Mindanao, Philippines, saw an increase of abaca (*Musa textilis*) plantations from 2,499 ha to 108,820 ha [77]. Abaca was commercially used for the production of fibers, also known as Manila hemp, a commodity in worldwide demand at that time. However, the expansion of abaca cultivation had serious consequences for the endemicity of wuchereriasis in the region, because the plant provides perfect breeding conditions for the vector of *W. bancrofti* on the Philippines, the mosquito *Aedes poecilus*. As a result, the prevalence of wuchereriasis increased substantially, which was shown to be positively correlated with the increase in the abaca cultivation area [77].

The guinea worm *Dracunculus medinensis* (Table 3) is another parasite that was brought to the New World by enslaved Africans [78]. This has been clearly documented in several accounts from the seventeenth and eighteenth centuries. However, as *D. medinensis* requires very specific environmental conditions and human behavior for local transmission, the parasite could establish itself only in a few places in tropical America for a limited period of time. With the abolition of the slave trade by the Spanish in the 1860s, no more local transmission of the guinea worm was recorded in Latin America. The occasional cases of dracunculiasis brought by immigrants and travelers were not enough to establish a chain of local transmission.

The rat lungworm *Angiostrongylus* (*Parastrongylus*) *cantonensis* (Table 3) is an example of a parasite that has been introduced into new areas by the spread of its definitive host. The worm is the etiological agent of eosinophilic meningitis in humans. The home range of *A. cantonensis* is thought to be in southern Asia where several genera of rodents, with *Rattus norvegicus* and *R. rattus* being the most important species, serve as definitive hosts [79,80]. Humans get accidentally infected with *A. cantonensis* when eating undercooked intermediate hosts (snails and slugs) or paratenic hosts (fish, frogs, and freshwater prawns) harboring infectious L3 larvae, or when consuming vegetables contaminated with snail and slug mucus containing the larvae. After ingestion, L3 larvae enter the brain where they grow into young adult worms. In rodents, the adult worms leave the brain and end up in the lung while in humans they remain in the brain causing eosinophilic meningitis. Since the end of the Second World War, *A. cantonensis* has been dispersed throughout Southeast Asia and Western Pacific Islands, including Australia [79]. The spread of the parasite was most likely via infected rats transported on ships and airplanes, and via the introduction of some species of snail, in particular the African land snail *Achatina fulica* [79]. Meanwhile, *A. cantonensis* is endemic in some Caribbean islands, south-eastern USA, Egypt, Nigeria, Côte d’Ivoire, Brazil, and Ecuador [80]. In addition, increasing numbers of travelers infected with *A. cantonensis* returning from endemic regions have been reported in Europe [81].

## Ectoparasites

### Mosquitoes

**Figure 4.**
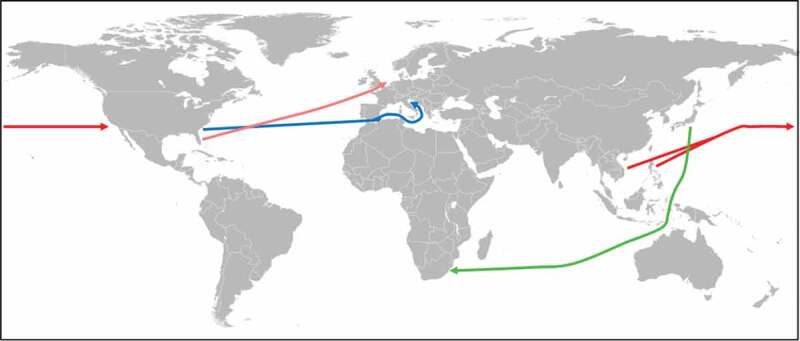
Spreading of *Aedes* sp. via international tire trade.

The Asian tiger mosquito *Aedes albopictus* (Table 4) is a well-documented example of the global spread of an ectoparasite through international trade in the twentieth century. Besides being a significant biting nuisance, *A. albopictus* is also a serious health risk as vector for chikungunya virus, dengue virus, and dirofilariasis (*Dirofilaria immitis* and *D. repens*). Originally, *A. albopictus* was native to the forests of Southeast Asia [82]. From there, the mosquito spread eastwards to Japan and South Korea and westwards to Madagascar, but did not immediately reach mainland Africa [83]. The first documented introduction of *A. albopictus* into the USA was in Los Angeles in 1946 and a second in Oakland in 1971 [82]. In both cases, the introduction of the mosquito could be traced back to the import of car tires from the Philippines and Vietnam, respectively (Figure 4). It should be pointed out that the natural breeding habitats of *A. albopictus* are small, restricted, shaded bodies of waters like water-filled tree holes, leaf axils, and rock pools, and thus man-made objects like jars, car tires, and tin cans provide acceptable alternatives. The mosquito was introduced a third time in Memphis in 1983, but the introduction route remained unclear [82]. However, all three introductions failed to establish *A. albopictus* in the USA. The first autochthonous occurrence of *A. albopictus* was recorded in Harris County, Texas, in 1985 [82]. As breeding places, car tires and other vessels were identified, which were also suspected as the vehicles for the introduction of the mosquito. It should be mentioned that between 1978 and 1985, the USA imported 11.6 million used car tires, two-thirds of which were from *A. albopictus* endemic regions [84]. Whether Harris County was the starting point for the expansion of the mosquito northwards and eastwards to other regions in the USA remains unclear. However, the spreading was not a natural expansion process as the maximum flying distance of *A. albopictus* is about 300 m/d [85], and thus it would have not been possible for the mosquito to reach the east coast within 2 y. More likely is that the mosquito was spread across the country through the trade with used car tires [82]. In addition, it cannot be ruled out that the occurrence of *A. albopictus* in different regions of the USA was due to repeated introductions of the mosquito [82]. The first evidence of *A. albopictus* in Latin America was recorded in the State of Rio de Janeiro in 1986 [86]. Subsequent detection of the mosquito along the Brazilian east coast initially suggested that *A. albopictus* may have been brought into Brazil from the USA. But this seems unlikely as the characteristics of the *A. albopictus* populations in Brazil indicated a different origin [82]. In the following decades, the mosquito spread throughout Latin America and is currently endemic in 19 countries [87]. In Europe, *A. albopictus* was discovered for the first time in Albania in 1979 [88]. The mosquito was probably introduced with a shipment of goods from China in the mid-1970s [88]. The next European country that became infested with *A. albopictus* was Italy. The mosquito was first detected in Genoa in 1990 [89]. In 1991, the first breeding population of *A. albopictus* was discovered in Padua in the Veneto Region (Figure 4) [90]. It seems very likely that the mosquito was brought into Italy with imported used car tires from Atlanta, Georgia, USA [91]. In the following years, the mosquito has become established in most regions in Italy below 600 m and the country is now the most heavily infested territory in Europe [92]. Since the millennium, *A. albopictus* has been introduced into many European countries, in most of which the mosquito has become established [92]. Besides the introduction via imported used car tires, the mosquito has been trapped along motorways indicating that it is spread in Europe via road traffic [92,93]. An alternative pathway of the introduction of *A. albopictus* into the Netherlands and Belgium has been by the import of Lucky bamboo shipments from China [94,95]. In Africa, *A. albopictus* has been reported for the first time in South Africa in 1990 (Figure 4) [96]. Once again, it was found that the mosquito was introduced with imported used car tires, but this time from Japan [96]. Although *A. albopictus* did not become established in South Africa, the mosquito has meanwhile colonized several other African countries [92]. Initial breeding sites were found in harbors and coastal areas indicating that the insect was probably spread by international shipping trade. The introduction of *A. albopictus* into Cameroon was most likely due to imported used car tires [97].

**Table 4.** Information about insects spread by human migratory activity.

Name	Origin	Current distribution
**Culicidae**		
*Aedes albopictus*	Southeast Asia	worldwide
*Aedes aegypti*	Africa	worldwide in tropical and subtropical regions
*Aedes atropalpus*	eastern North America	North America, Europe
*Aedes japonicus*	East Asia	East Asia, North America, Europe, New Zealand
**Reduviidae**		
*Triatoma rubrofasciata*	South America or Asia	Americas, Asia, Africa, Oceania
**Pulicidae**		
*Tunga penetrans*	South America	Central and South America, sub-Saharan Africa

The first introduction of *A. albopictus* into the USA (Los Angeles and Oakland in California) could be traced back to a shipment of used car tires from the Philippines and Vietnam (red arrows) [82]. The establishment of *A. albopictus* in Padua, Italy, could be linked to used car tires imported from Atlanta, Georgia, USA (blue arrow) [91]. The first reported case of *A. albopictus* in South Africa could be attributed to a consignment of used car tires from Japan (green arrow) [96]. The introduction of *A. aegypti* into the Netherlands could be tracked to a shipment of tires from Miami, Florida, USA (orange arrow) [103].

*Aedes aegypti* (Table 4) is another mosquito species that had been spread by human activities in the past. The mosquito poses a serious health risk as vector for the yellow fever virus, dengue virus, chikungunya virus, and Zika virus. Nowadays, it is one of the most widespread mosquito species in tropical and subtropical regions [98]. Historically, *A. aegypti* has been dispersed throughout the world on sailing ships from Africa [98], the original distribution range of the mosquito. Seventeenth-century reports on dengue-like epidemics (note that *A. aegypti* is the main vector for Dengue) in the Caribbean suggest that the mosquito was probably already introduced into the Americas with the onset of the slave trade [99]. From the late eighteenth to the mid-twentieth century, *A. aegypti* became established in Southern Europe [98]. Unlike *A. albopictus, A. aegypti* has not extended its distribution range in Europe beyond the Mediterranean because the eggs of the mosquito are unable to undergo winter diapause [98]. During the second half of the twentieth century, *A. aegypti* has disappeared from many foci in Europe and America. The reasons for its disappearance are not fully understood but eradication programs have contributed to the reduction of *A. aegypti* in South America between 1947 and 1970 [99] and the global spread of *A. albopictus* since the 1970s may have resulted in the competitive displacement of *A. aegypti* [100,101]. More recently, however, *A. aegypti* is recolonizing Europe via modern transport systems (air and road travel) [98]. In the summer of 2010, the mosquito was discovered in the Netherlands at tires yards [102]. Genetic analysis revealed that *A. aegypti* was introduced via a shipment of tires from Miami, Florida, USA (Figure 4) [103]. This finding was quite unusual as the spread of *A. aegypti* is not directly associated with the international trade in used tires [104].

Two more *Aedes* species have been spread around the world by commercial transport of used tires. *Aedes atropalpus* (Table 4) is a native North American mosquito species that has been introduced into Europe several times between 1990 and 2009 [105]. Although *A. atropalpus* is reproducing in Europe, the established populations have remained localized so far [105]. *Aedes japonicus* (Table 4) is endemic in East Asia and has been spread since the 1990s [106]. The mosquito was first reported outside its native range in New Zealand in 1994 [107]. In 1998, *A. japonicus* was discovered in the north-eastern USA [108] and the spread of the mosquito within the country may have been facilitated by the Standardbred horse trade [109]. Since 2000, *A. japonicus* has been present in Europe and established in western regions of Germany [106]. However, both mosquito species are not considered important vectors for diseases.

### Kissing bugs

The tropicopolitan distribution range of the triatomine species *T. rubrofasciata* (Table 4), a vector for *T. cruzi* in Latin America, can be only explained by recent spreading events. DNA sequence analyses using nuclear and mitochondrial marker genes showed high similarity between New World and Old World specimens indicating a common and recent origin of Asian and American populations of *T. rubrofasciata* [110,111]. The close association of *T. rubrofasciata* with domestic rats (especially *R. rattus*) suggests that the triatomine bug was spread around the globe by international shipping during the sixteenth to eighteenth centuries [112]. However, the place of origin of *T. rubrofasciata* is still debated. One hypothesis suggests that *T. rubrofasciata* originated in the New World and could be the common ancestor of other Asian triatomines [42]. An alternative hypothesis proposes that the triatomine bug is of Asian origin and was recently introduced into the Americas [42]. Meanwhile, *T. rubrofasciata* has become a serious biting nuisance and a public health problem in Vietnam as bites by the insect can produce severe anaphylactic reactions in humans [42,111]. Reports of people bitten by the triatomine bug have significantly increased in different Vietnamese cities over the last decade [111]. The reason for the current widespread infestation of *T. rubrofasciata* in urban areas in Vietnam is unclear. One explanation could be the massive culling of peridomestic chickens in urban and periurban areas in the previous decade in order to control avian influenza. Triatomines readily feed on chickens, which in rural Latin America have been shown to be important for the bug's transition from sylvatic to domestic lifestyles. Thus, it seems that human interference led to the loss of a primary host of *T. rubrofasciata* driving the triatomine bug to prey on people [42].

### Sand fleas

The sand flea *Tunga penetrans* (Table 4) is one of the few examples of a parasite that has been spread from the New World to the Old Word. Only fertilized female fleas become skin parasites and burrow into the stratum granulosum of the epidermis. In contrast, *T. penetrans* larvae live in sand and soil while adult sand fleas feed intermittently on their host. Originally, *T. penetrans* was a parasite of Xenarthra (armadillos, anteaters, and sloths) and/or Carviidae (guinea pigs and capybaras) in the neotropics [113]. With the arrival of *H. sapiens* in Central and South America, the flea adopted humans as an additional host in pre-Columbian times [114]. The parasite expanded its host range further to include domestic animals and rodents introduced by Europeans during the colonization of America. The introduction of *T. penetrans* into Africa can be traced back to a single event. In September 1872, the flea was brought from America to Africa with the English ship “Thomas Mitchell” that sailed from Rio de Janeiro to Ambriz in Angola [113–115]. With ballast sand, old coffee bags, infected sailors, and people visiting the ship, the parasite got ashore. First, the flea was dispersed along the coast by shipping active in those days. Within a few years, the parasite was spread eastwards and southward along trading routes with traders, explorers, and soldiers, and by 1888 and 1890 had already reached Mozambique and Natal, respectively [113]. By the end of the nineteenth century, sea trade had brought the flea onto the islands off the African east coast (Madagascar, Zanzibar, Seychelles, Comoros, Mauritius, and Reunion). In 1899, British soldiers introduced *T. penetrans* into the Indian subcontinent but the flea never became established there [115]. Meanwhile, *T. penetrans* has established itself in most sub-Saharan countries.

## Concluding remarks

The human tendency to explore and colonize new areas has largely contributed to the spread of parasites. The different forms of voluntary migration (emigration, immigration, external and internal migration, labor migration) were and still are one of the main causes for the dispersal of parasites throughout the world. The transatlantic slave trade, which can be regarded as a special form of forced migration, played an important role for the introduction of many new parasite species into the Americas. Displacement and relocation of people caused by war and civil unrest are also types of forced migration that have led to the spread of parasites up to the present day. Trade, in particular shipping, has been and still remains a very effective mode of spreading parasites around the world. More recently, air and road transport have also contributed to the spread of parasites. In addition, through modern mass tourism, parasites have been repeatedly introduced into non-endemic areas, in most cases without consequences for the range expansion of the species involved.

It has been predicted that climate and environmental changes will affect the geographical distribution of parasites and their human hosts [116–118]. Global warming should facilitate the establishment of parasites and disease vectors into more temperate parts of the world as their optimal temperature for development will shift northwards [116]. Increased precipitation and humidity should favor parasites that rely on aquatic and free-living life cycle stages (e.g. mosquitoes and soil-transmitted helminths, respectively) [116]. Environmental changes (e.g. deforestation) can create novel habitats for parasites that can help to establish them in new areas [116,119]. Importantly, climate change may cause massive migrations as some areas may become uninhabitable through droughts, an increasing problem particularly in sub-Saharan Africa [117]. It can be expected that migrants will carry parasites and introduce them into new regions [117]. However, it is quite difficult to predict what overall impact anthropogenic climate change will have on the spread of parasites in the future.
